# Factors affecting the quality of endodontic treatment in general dental practice in Scotland: a qualitative focus group study

**DOI:** 10.1038/s41415-022-4475-4

**Published:** 2022-07-22

**Authors:** Sonia Khamuani, Alastair Ross, Douglas Robertson

**Affiliations:** University of Glasgow, Glasgow, UK

## Abstract

**Introduction** The quality of endodontic treatment worldwide is variable despite clear clinical guidelines being available.

**Aims** Identify the factors that affect the quality of endodontic treatment in general dental practice in Scotland and how to overcome the barriers identified.

**Methods ** Four online focus group interview sessions were conducted in which a total of 16 participants were recruited, including general dental practitioners, endodontic specialists and educationalists. Topics for facilitated discussion were based on healthcare system theory and included education/training, patient factors, resources and equipment factors; themes were identified via template analysis.

**Results** A number of themes and subthemes affecting the quality of care are reported, including aspects to do with remuneration, time, education, training, support from secondary care and the impact of COVID-19.

**Conclusion** Findings support: increase practical experience for undergraduates; access to postgraduate mentoring and training; development of a managed clinical network to facilitate access to appropriately trained specialists; and increased remuneration to allow sufficient time and adequate equipment to achieve clinical standards.

**Supplementary Information:**

Zusatzmaterial online: Zu diesem Beitrag sind unter 10.1038/s41415-022-4475-4 für autorisierte Leser zusätzliche Dateien abrufbar.

## Key points

- Despite available clinical guidelines, the quality of root canal treatment in NHS general dental practices is suboptimal.
- A variety of factors influence the quality of treatment in general dental practices, such as cost of treatment and equipment, a lack of training or education, inadequate support from secondary care and the impact of COVID-19.
- Adequate funding and support for clinicians could allow them to invest appropriately in treatment and equipment to improve the quality of care in NHS.
- Continuing professional education is frequently seen as a tool to improve the quality of care.

**Supplementary Information:**

Zusatzmaterial online: Zu diesem Beitrag sind unter 10.1038/s41415-022-4475-4 für autorisierte Leser zusätzliche Dateien abrufbar.

## Introduction

Endodontic treatment is concerned with the management of infection and inflammation within the pulp and periapical tissues of the teeth and is perhaps one of the most technically demanding aspects of dental care. Endodontic treatment accounts for a significant amount of NHS dental spending, with 109,881 claims for endodontic treatment in Scotland in the NHS general dental services in 2019. Most of the endodontic treatments were carried out in primary dental care. Clinical guidelines have long been established in endodontics and success in specialist settings is high but, despite this, the quality of care provided in general dental practice across the world appears to fall short of these standards. A number of epidemiological studies have reported that the prevalence of apical periodontitis was between 25-50% in endodontically treated teeth and this was shown to be due to inadequate or substandard root canal fillings.^1^^,^^2^^,^^3^ Kirkevang *et al*.,^2^ while assessing the periapical status of endodontically treated teeth in the Danish population, found that teeth with inadequate root canal filling and poor coronal seal were associated with apical periodontitis. The study also reported the presence of apical periodontitis in approximately half of the endodontically treated teeth, that is 52.3% out of 773 teeth. Despite advanced technology and instrumentation which have simplified the procedure, the quality of endodontic treatment in general dental practices is still compromised and the radiographic appearance of root canal filling are suboptimal.^4^

Limited data are available to explain the rationale for not performing adequate endodontic treatment in general dental practice in the NHS. Previously, McColl *et al*.^5^ investigated the barriers to improve endodontic care in the NHS in the UK. The study collected responses from dental practitioners and it was identified that the main barriers which compromise the quality of care in the NHS are remuneration and lack of education or training. Dentists reported that the amount of time required to perform good-quality treatment is not in accordance with the provided money. Furthermore, it has been observed that general dental practitioners (GDPs) find root canal treatment challenging and difficult. The emotions of frustration, stress, anxiety and exhaustion have been found while undertaking root canal treatments.^4^ Studies have established certain barriers in general dental practice which compromise the quality of the treatment. The root canal treatment performed in general dental practices is not ideal and there is room for improvement in the technical quality of endodontic treatment in the NHS.

Given the high success in hospital settings (even when these include undergraduate students)^6^ and the clear guidelines on how to perform adequate endodontic treatment, it is possible that the reasons for the suboptimal outcomes in primary dental care are not as a result of technical skill or knowledge but rather are as a result of issues within the system that mitigate against achieving good outcomes in endodontic treatment. The assessment of quality in healthcare is a complicated and complex procedure; it requires understanding of individuals' perceptions and experience.^7^^,^^8^

## Aims

- Identify the factors that affect the quality of endodontic treatment in general dental practice in Scotland
- Gather information on how to overcome these barriers to improve the quality of root canal treatment in Scotland.

## Design

This was a focus group study involving qualitative exploration of factors affecting the quality of endodontic treatment in clinical practice. Data were analysed using template analysis^9^^,^^10^ which is a form of thematic analysis which allows interpretation of textual data in a structured form using *a priori* themes (the 'template') to guide coding, while still allowing flexibility and coding of emergent findings.^11^

### Procedures

#### Recruitment

Non-probabilistic purposive sampling was employed where participants were approached due to their ability to inform the aims of the research.^12^ An invitation email with participant information was sent in April 2021 to all dental practitioners, endodontic specialists, dental trainees and educationalists through Glasgow Dental School (where posters were also displayed), the local NHS Oral Health Directorate, Glasgow Odontological Society and the Scottish Dental Network. Interested participants were told the study was voluntary and that they were at liberty to withdraw at any time up until the focus group transcripts were anonymised. A signed consent form was collected from all the participants who agreed to proceed with the study. All participants had the opportunity to re-review the participant information sheet before the focus group session. The number of focus groups was finalised based on principles of data saturation, where no new themes emerged.^13^ Participants are shown in Table 1.

**Table 1:** List of participants involved in the study

**Focus group 1**	**Focus group 2**	**Focus group 3**	**Focus group 4**
Participant 1 (GDP) (M)	Participant 4 (GDP) (F)	Participant 9 (educationalist) (M)	Participant 14 (GDP) (F)
Participant 2 (GDP) (M)	Participant 5 (GDP) (M)	Participant 10 (educationalist) (M)	Participant 15 (GDP) (M)
Participant 3 (endodontic specialist) (M)	Participant 6 (GDP) (M)	Participant 11 (specialist) (M)	Participant 16 (GDP) (M)
	Participant 7 (GDP) (M)	Participant 12 (specialist) (M)	
	Participant 8 (GDP) (M)	Participant 13 (specialist) (M)	
Key: P = participant F = focus group M = male F = female			

#### Facilitation

Focus groups lasted approximately 60 minutes and were conducted online using Microsoft Teams, in line with local and national restrictions on non-essential travelling and contact during the COVID-19 pandemic.^14^ Microsoft Teams allowed reliable and secure real-time audio and visual recordings of discussions involving multiple participants.

A topic guide was developed and tested in a pilot focus group overseen by an experienced qualitative researcher/psychologist.^15^ Pilot participants were asked to provide their thoughts on facilitation and topic guide/questions which were modified in accordance with feedback. Data from the pilot group were not included in analysis.

General topics for facilitated discussions were: participant background; assessment of quality of endodontic care from participants' perspectives; barriers to optimal endodontic treatment in practice; time, equipment, resources and remuneration; undergraduate and postgraduate training; patient factors and expectations; evidence-based practice (for example, rubber dam); and impact of COVID-19. Prompts and rephrasing of questions were used as appropriate during the session by the researcher to explore topics and gain insight from participants.^1^^6^ All focus group interviews were moderated by the first author (researcher) along with the experienced clinician (third author) except the fourth focus group (GDPs only) which was solely moderated by the researcher themselves.

### Data security

A Data Protection Impact Assessment was carried out in accordance with university ethical approval (Research Ethics Committee number: 200200076). Data were stored on a password-protected server in compliance with UK General Data Protection Regulation (2018). Transcripts were anonymised, with names and personal information redacted and stored on the University of Glasgow's secure OneDrive. The data collected in the study were only accessed by the research team and no data were shared with any external parties.

### Analysis

An automated transcript was generated using Microsoft Teams and the encrypted files were converted into a Word document using RStudio software for manual correction and data analysis. This process of manual correction also allowed the research team to become familiar with the collected data.

**Fig. 1:**
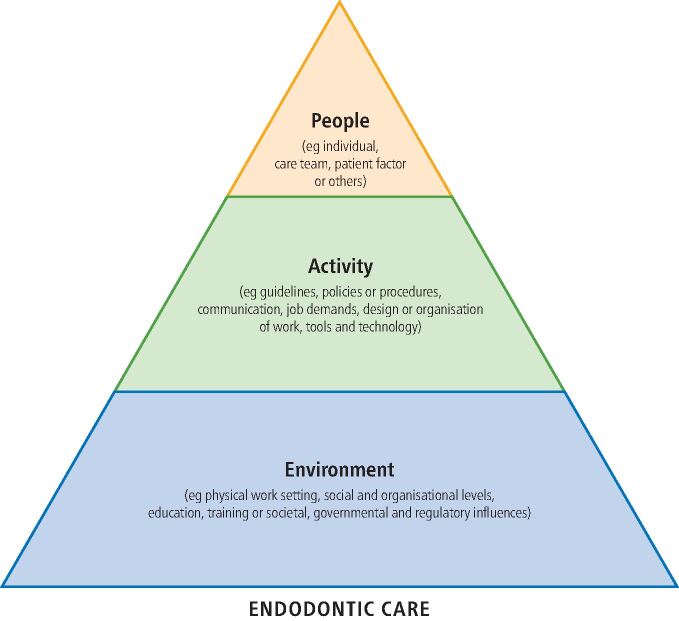
The NHS Education for Scotland template used for data analysis (adapted with permission from PAcE worksheet, NHS Education for Scotland on the Turas Learn platform, 2021)^17^

The initial template for analysis (Fig. 1) was the People, Activity, Environment (PAcE) model published by NHS Education for Scotland.^16^ This frame was developed for primary care to enhance significant event analysis and was employed to ensure that system factors at all levels, from individual through to organisational, could be explored.

After coding through the model, emergent themes and subthemes were extracted and discussed within the research team. In summary, steps in accordance with template analysis guidelines^9^ were:

- Manual editing and familiarisation with transcripts
- Template used to code data based on *a priori* categories
- Interpretation of codes to generate emergent themes and subthemes.

## Results

Study participants are shown in Table 1.

### Quality of care

Quality was thought in general to be good but GDPs were facing several barriers in terms of patient and staff factors, task and resources and the wider environment. As one participant said:

- '*I wouldn't say the standards are like excellent. They are done probably to the best of our abilities in the timeframe we have and with the equipment available*' (p1f1).

These factors are outlined in the online Supplementary Information, which show the PAcE analysis. The main factors affecting care are now presented as integrative themes which draw from this coding. Further illustrations are shown in the online Supplementary Information.

### Practice leadership and management in the context of time and financial pressure

Participants reported that the culture set by the practice owner/principal is important and can be a barrier to optimal endodontic treatment if there is more of a focus on financial considerations rather than the quality of treatment provided *per se*. Principals have an influence on how long the associates are allowed to spend on cases, the quality of materials and the availability of equipment. As one participant reports:

- '*I think that my associate colleagues are compromised and it's very difficult because sometimes principals have got more of an eye on the bottom line of the accounts rather than the quality*' (p1f4).

### Evidence-based tools and equipment

Related to this is a reported tendency to use rotary for private treatments and hand files for NHS based on the expense of the treatment. Rotary is expensive and is said to improve treatment quality. Similarly, lack of magnification, which also comes with significant cost, affects the quality of treatment, as one participant puts it:

- '*It's very difficult to be like troughing if you can't see what you're doing so you know, some pieces of equipment are of limited use if you don't have magnification*' (p3f1).

Reported use of rubber dam was variable and reluctance was more attributed to skills/confidence than cost *per se*. Some participants mentioned that its use could be 'actively discouraged'.

### Education and training

Findings suggest that training at undergraduate and postgraduate level is adequate to carry out good-quality treatment in straightforward root canal treatments. There was less agreement on whether further training is necessary and on what form that this 'continuing development' training should take. One person felt this was appropriate for those with a 'special interest':

- '*If I want to set myself up as a dentist with special interest in endodontic then absolutely, I need to keep up to date so I need postgraduate training from that point of view*' (p2f1).

As might be expected, recent graduates are reportedly less confident in carrying out endodontic treatments due to a lack of volume of cases completed during undergraduate training; however, as intended by the General Dental Council curriculum, they are perceived to have sufficient core knowledge to undertake simple root canal treatments as a 'safe beginner'.

### NHS remuneration

Related to the themes above, there is a more general thematic discussion about NHS remuneration. Most participants were very unsatisfied with the remuneration for NHS-subsidised root canal treatment, which was termed 'a joke'. The GDPs mentioned that there has been no change in remuneration under the NHS for some time:

- '*I qualified in 2001 and what we get paid since then hasn't really increased in line with the increasing cost of everything else or equipment, rates, electricity, wages etcetera*' (p4f2).

The participants generally agreed that the amount of money that dentists get is not adequate for the time that a good-quality endodontic treatment requires. An example would be if a GDP found an extra canal in a tooth, or if a case led to complications, whereby there would be no commensurate increase in remuneration.

### Secondary care and referrals

The informants felt that the referral system in the NHS is difficult and time-consuming, with insufficient resources available to support GDPs with complex endodontic cases. In addition, an important consideration is that patients may also prefer treatment by their GDP. Consequently, GDPs may undertake more complicated root canal treatments than otherwise optimal, which compromises the treatment quality.

### COVID-19

As might be expected, the COVID-19 pandemic and response has affected many aspects of care.

Clinical impact included the loss of teeth which were planned for root canal treatment before 'lockdown' but became unrestorable and were subsequently extracted. Some teeth were accessed and opened for treatment during the pandemic which was never completed.

The practitioners felt that it was difficult to perform aerosol generating procedures (AGPs) in general dental practice during COVID-19. Many patients opted for extraction of a tooth instead of root canal treatment due to lack of accessibility to AGPs and personal protective equipment. There were also difficulties in taking radiographs under AGP requirements.

Referrals reportedly increased; however, there was a change in acceptance criteria due to limited resources available in health services and only patients with predictable prognosis were selected for the treatment. Not all responses were negative, however, and one respondent talked about executing thorough process:

- '*It's just part of being the same process really, I think with COVID-19 being a bit more thorough with our kind of working environment*' (p16f4).

### Suggestions for improvement

A final theme was that participants felt there was scope to improve the quality of care in general practice. Funding was of course mentioned, both in terms of basic fees ('*quadruple the fee so that I can spend longer doing it*' [p2f1]) and for equipment. It was felt that higher fees would serve to raise standards. Specialist practices with those trained to a higher/enhanced level was suggested. A managed clinical network was suggested to support GDPs by allowing them to discuss management of endodontic cases with an accessible and flexible support network of professionals with appropriate training.

## Discussion

This small focus group study found several barriers to the provision of quality endodontic treatment. Qualitative self-reports are always subject to demand characteristics of the situation (in this case, having clinically trained researchers recording discussions) but there was a fair consensus among respondents and no reason to believe any answers were withheld or not given freely. A key issue is remuneration in the NHS, which is related both to the amount of time available to carry out the treatment and the feasibility of purchasing the necessary equipment and consumables. Similarly, McColl *et al*.^5^ found that remuneration is also a key barrier in NHS general dental practices in England and called for NHS fees for endodontic treatment to be revised.

Studies have previously revealed that there is lack of rubber dam use in general dental practices which results in inferior endodontic treatment outcomes.^18^^,^^19^^,^^20^^,^^21^ Whitworth *et al*.^22^ reported infrequent use of rubber dam in 2000 and suggested that the NHS fee for root canal treatment was not adequate to justify rubber dam use and its placement requires time, which is at a premium in NHS dentistry. In contrast to this, only a few participants admitted to not using rubber dam in the past, which they reported was due to lack of availability and working in an unsupportive dental environment. This may reflect recent reports that usage has increased.^23^

Motorised endodontic systems allow a clinician to perform endodontic treatment more efficiently with fewer procedural errors and heated obturation systems help obtain a higher quality root canal filling.^24^ These are relatively expensive and this could be a barrier; however, other participants felt that the improved efficiencies offset the initial cost. If equipment was funded more specifically by the NHS, then this could help encourage practice principals to invest.

Education and training also have a crucial impact on the quality of care provided in general dental clinics. It was stated that due to the low volume, the recent graduate often lacks confidence and is less competent to perform root canal treatments in dental practices. These findings align with those of other recent studies.^6^^,^^25^ Some participants felt that postgraduate training is necessary to improve practitioners' skill in root canal treatment; however, other participants emphasised the importance of continuing professional education, as it has been reported that post-graduate training and accreditation is for those who wished to provide a more specialist level of care in complex cases.^26^

GDPs in the study reported having difficulties in accessing specialist level assistance with difficult cases in general and these challenges have increased during the current pandemic^27^ with a reduction in capacity in the secondary care services and limited acceptance criteria. A lack of access to specialist level assistance meant that the GDP is faced with attempting treatment that they are not confident in completing successfully, or extracting the teeth. Increasing the number of high street specialists and practitioners with enhanced skills would improve access for more complex cases.

## Conclusion

In conclusion, allocation of sufficient time and government investment in the appropriate equipment and training for dentists in the general dental service is indicated as a priority. Further work is required to develop increased payments for practitioners with enhanced skills and access to specialist services within the community and secondary care services for cases with additional complexity.

## Supplementary Information

Supplementary Information (PDF 127KB)
