# Supplementary material for: Factors affecting the quality of endodontic treatment in general dental practice in Scotland: a qualitative focus group study
Source: Br Dent J. 2022 Jul 22;233(2):129–33. doi: 10.1038/s41415-022-4475-4 (PMC9305044; doi:10.1038/s41415-022-4475-4)

Supplementary File 1 Integrative themes and examples of important factors for endodontic care

Theme	Description	Illustration
**Practice leadership and management in the context of time and financial pressure**	Principals' allocation of time and material	"*I was given an hour to do molar with root canal treatment which I think was quite generous compared to some of my colleagues, but it probably made it not a very lucrative treatment.*" (p5f2)<br>"*Some principals would say well use hand files, use your k files because they're cheaper, don't use the fancy DENSPLY file ones or whatever[..] it takes longer to use those types of files.*" (p4f2)
	Associates focusing on remuneration	"*Some of our associates are money oriented and they will say look I don't want to spend an hour and a half doing this for now […] I am going to try to do in a much quicker way*"(p16 f4)
**Evidence-based tools and equipment**	Rotary systems are viewed as optimal, but costly	"*Obviously, the instrumentation is only one facet of endodontic treatment. I think it makes it efficient. It makes it […] reproducible. […] the thing is that your shape is fairly consistent when using a Rotary driven system.*" (p3f1)<br>"*I think is it sometimes practice owners could be little bit short sighted in investing [in equipment] because they don't think about the long-term effects of their investment.*" (p3 f1)

	Magnification is key but requires investment	“*I think magnification is the key thing, but the expense of magnification is significant […] if someone is making those investments in a practice it may be difficult to then retain them within the NHS*” (p12f3)
	Rubber dam can be difficult to master, and use is not universally encouraged	“*I don't think that they [GDP] had developed the skill to pass that confidence onto the patient so they end up having difficulty with rubber dam quite.*” (p11f3)<br>“*The first practice I worked in as a VT practice with a rubber dam kit […] It was actively discouraged and believe it or not when I moved practice 4 years later, I knew that this was something I should be doing, and I wanted to move to somewhere [I could use rubber dam]…*”(p6f2)
**Education and training**	Undergraduate education provides for ‘safe beginners’ as intended, but case experience is somewhat limited	"*I agree with the point about experience makes you better at it and learning makes you better as well.*" (p6f2)<br>“*I think the barrier we have for undergraduate teaching is the amount of exposure they get to cases*” (p4f2)

	Lack of consensus on the level of postgraduate training is required	“*I think to undertake a good quality endodontic treatment people should have postgraduate training. There are challenges right from the point of sort of diagnosis, all the way through to every single element of how the procedures carried out and I think it's one of the most complex procedures in dentistry*.” (p12f3)<br>“*I think training targeted to [the] level which the profession is going to be performing […] there are clearly different complexity in endodontic treatment*” (p13f3)<br>“*I don't necessarily agree that postgraduate training is necessary to provide a good quality endodontic treatment, I think undergraduate training provide a competent safe beginner*” (p9f3).<br>“*I don't think you need to go out, get postgraduate training but you're always needing to update everything anyway, that's the case for everything*” (p14f4)
	Further training is indicated for those with a special interest	“*If I want to set myself up as a dentist with special interest in endodontic then absolutely, I need to keep up to date so I need postgraduate training from that point of view*.” (p2f1)

**Time and remuneration in NHS**	Remuneration is inadequate	"*[…] I think the biggest kind of factor would be remuneration in NHS I think that's where people might think well if I'm not getting that much how can I maximize my intake.*" (p16f4)<br>"*[…] With regard to the time constraint, that's a huge issue and the financial reward for doing it is in my considered opinion appalling for what they're (NHS) expecting us to or what we are taught as undergraduates […] the financial renumeration is just a joke, have to be brutally honest with you.*" (p15f4).<br>"*Sometimes in the [NHS] if you find an extra canal that makes your job more difficult […] you have to give more time to it but you're still getting remunerated in the same way which is quite significant*" (p1f1)<br>"*As soon as you get into any kind of more complex stuff, it takes more time, that's an issue[...] I would say that there is only your own clinical wanting to do that. The government don't really kind encourage quality.*"(p6f2)
	Difficult referral process	"*I had cases that are without a doubt specialist cases that have been rejected […]*" (p6f2)<br>"*I think that referral system has become more difficult […]*" (p16f4)

**Secondary care and referrals**	Relationship with patient	"*[…] you end up taking cases that you may think that slightly complicated and which will affect your outcome…. You're not going to get the outcome you want but your patient really wants the treatment and it's that relationship you have with the patient.*" (p1f1)<br>"*You have been seeing a patient for 10 or 15 years and built up a relationship with them and they are perhaps less inclined to go elsewhere for treatment.*" (p1 f1)
**COVID-19**	Interrupted treatment plans	"*I have lost teeth as a result of COVID as somebody had a retreatment, I cleaned and got it symptomless, this was in February March last year but then he attended [after lockdown] the tooth had split, and I took it out in August.*" (p6f2)<br>"*A lot of teeth have obviously been opened when I worked at the PDS centres opening teeth but then probably not picked up treatment from there and when it has got that next stage the tooth actually not being fixable at that point.*" (p7f2)
	AGPs	"*It was difficult to actually get that aerosol procedure at that point and you had to have that discussion as to "do we hold off and wait or do we just manage it now with extraction? And it was patient led but probably more patients said yes for extraction.*" (p6f4)<br>"*The only thing was taking radiograph that you have to open the doors from AGPs, step outside take radiograph […] while the other people rooms were big enough to stay in the room*"(p1f1)

	Referrals	"*There have been a lot more referrals, since the pandemic has ended whether that does reflect a change […] we're seeing patients may be at a more advanced stage of disease that need specialist care*." (p3f1)<br>"*[…] we have slightly modified our acceptance criteria […]in health service we can only really accept teeth where it's likely that the outcome would be predictable*." (p13f3)

Supplementary File 2 Coding from adapted PAcE template: people factors

PAcE category	**Subtheme**	**Illustration** (participant; focus group)
**Individual**	The quality of endodontic treatment improves with experience of an individual	"*I agree with the point about experience makes you better at it and learning makes you better at it as well*." (p6f2)<br>"*I'm sure have been doing this long enough to assess which root treatments are appropriate for me to do*" (p2f1)
	Recent graduates lack confidence and experience	"*They've obviously they've done it enough to be safe beginners but that's all they are at that point. […] it's inevitable that it's going to take a while for them to build their confidence and all.*" (p3f1)
	Individual who are interested in endodontics will spend extra time to perform good quality treatment	"*I think the ability of the dentist and some dentists are highly skilful and interested in root canal treatment and some aren't. I would say it is down to individual ability*" (p10f3)<br>"*There is a wee bit about skill enhancement and people's interest in it" [endodontic treatment]*" (p10f3)
**Care team**	The leader of the practice could be a barrier because they focus more on the amount of the money you are getting from the treatment rather than the quality.	"*It's just depending on the owners of the practice or If the dentist himself and is willing to fund that and subsidizing the treatment*". (p5 f2)<br>"*I think that my associate colleagues are compromised and it's very difficult because sometimes principals have got more of an eye on the bottom line of the accounts rather than the quality*" (p15f4)
	Inexperienced or incompetent staff increase individual stress levels which could led individuals to make mistakes in the treatment.	"*sometimes staff can be a barrier to…not to successful root treatment but to the whole picture surrounding your stress levels with regards to successful root treatment, whether or not your nurses is competent at what they should be[..] but I don't think any of these things would change about how I would do it, I just feel under more stress*" (p4 f2)

**Patient factors**	Positive outcomes of endodontic treatment in general dental practice arise when no complications emerge, besides quality is compromised when complication arises.	"*If you don't come across a complication and everything proceeds as planned generally you tend to get a good result*". (p1f1)<br>"*If everything goes right first time. If it is easy, as most endo treatment are, it is pretty straightforward then*." (p2 f1)<br>"*As complexity increases quality potentially goes down*" (p9f3)
	Patients with complexity are less inclined to go for private specialists because of the relationship between dentist and patient.	"*You have been seeing a patient for 10 or 15 years and built up a relationship with them and they are perhaps less inclined to go elsewhere for treatment.*"<br>"*it's that relationship you have with the patient and its your patient and you're going to do because it's your patient and it needs to be done.*" (p1 f1)
	Patient preference and expectation re cost can be unrealistic	"*there's the patient view on expense and whether they're willing to be referred to a specialist endodontist which might be the most appropriate place for this to go but they want me to do it for 80 quid*." (p2 f1)<br>*"I think at times the patient's expectation of what can be done within the fee structure is perhaps unrealistic*" (p2 f1)
	Patients are more demanding, and expectations are high in general dental practice which puts pressure on dentists.	"*They are more demanding I think patients in general now and that their acceptance of when things go wrong is not so good.*" (p4 f2)<br>"*I think expectations have soared that obviously puts the huge amounts of pressure on us to be on our top game every time. "Actually, you know what, so we really need to look after ourselves as well because if we don't, we're only going to end up burning ourselves out trying to please people that are unpleasable*." [patients] (p15 f4)

Supplementary File 3 Coding from adapted PAcE template: activity factors

Subcategories	Description	Illustration
**Procedures**	All the steps involved in caring out root canal treatment are being followed by the dentists whether its NHS or private	"*In practice. We were practicing. We were doing most of the steps as per the protocols that were there like using rubber dams etc. (p8 f2)* *I have been following the best protocols or the same protocols whether it's in NHS or private treatment*." (p7 f2) "*I think probably trying to get the good outcome for patient can be quite challenging but with each place you work you have to try and figure a way through that and stick to the protocol you're used to yourself. you know the one you know that works.*" (p4f2)
	By using loupes, magnification for all the quality of endodontic treatment could be upgraded.	"*I think we should probably start looking at the minimum standards for provision of endodontic care being carried with the loupes I think that we should be wearing them. "We make almost mandatory for endodontic care*"(p3f1)
**Work processes**	The time provided for the treatment in workplace have a significant impact on the quality of care which is provided. There is not enough time to perform a good quality treatment under the health service	"*I was given an hour to do molar I was allowed to an hour half with root canal treatment which I think was quite generous compared to some of my colleagues and it probably made it not a very lucrative treatment.*" (p5f2) "*I would say the other issue with time is the tendency in NHS general practice to underestimate the time required [for treatment]*" (p9f3) "*It is mainly down to the excessive amount of time it takes to do a high-quality endodontic treatment possibly not [feasible] under the health service*" (p11f3)

	Time pressure indirectly affects practitioners' abilities to do a good job.	"*I think it will, it's almost bound to [individuals' attitude and willingness], probably not directly but it's bound to indirectly affect the person doesn't go out of their way trying to rush but other factors will mean that you are more likely to rush*" (p9f3)
**Job demands/organ ization of workplace.**	When dentists come across complications there are limitations to what can be done in general dental practice because of time, extra cost, and efforts required.	"*the way you are remunerated for the treatment, so you only have you only can devote X amount of time to the treatment, you can't come back to same tooth 3 or 4 times and trying to work on complications*" (p1f1)<br>"*[…] (Complex case) a tooth with an open apex: I did actually do it in the end, but I had to buy in extra equipment, but I bought that in at a loss to do this but there's no recognition of complexity*" (p6 f2)
	Difference in filing system in private and NHS based on the expense affects the quality of treatment	"*There are quite a few practices I've heard of that you're only allowed to use the Rotary endo for private treatment and for NHS treatment you have to use the hand files because they're cheaper so that's obviously going to be a barrier to success*" (p14f4)
**Tools and technology**	Rotary driven systems increase consistency and help to create better shape of the root canal.	"*Obviously, the instrumentation is only one facet of endodontic treatment. I think it makes it efficient. It makes it really […] reproducible […] The thing is that your shape is consistent when using a Rotary driven system.*" (p3f1)<br>"*But of course, we did have, you know other drawbacks as not having enough or the required kind of materials etc. Or the Rotary files were not available sometimes.*" (p8f2)

	Practices differ in the availability of rotary system and proper instruments that are required for adequate root canal treatment	“*I've had some of the situations as well Where you know certain principles don't allow rotary endo which is a shame because it's almost counterproductive because essentially you want an endo done well and efficiently*” (p16f4)<br>*“There's quite a few practices I've heard of that you're only allowed to use the Rotary endo for private treatment and for NHS treatment you have to use hand files because they're cheaper, that's obviously going to be a barrier to success.” (p14f4)*
	Rubber dam can be difficult to master, and it requires skills to put it on patients with confidence initially	“*When you're starting out with rubber dam, It is quite difficult to master. especially if you're trying to teach it to yourself during when you’re supposed to be a fully qualified dentist. If you try to put it on the field few times you feel silly in front of your patient*.” (p1f1)<br>*“It's definitely environment if it is supportive and encourages its [rubber dam] use. Then it will get used and it will become ingrained in your practice (p3f1)*
	The lack of magnification is a barrier in general practice which compromises the quality of the endodontic treatment	“*I think you can't quite get to the bottom probably don’t have the magnification or tools that maybe need to identify the fractured tooth that is unrestorable*” (p1f1)<br>“*[…] perhaps the certain barriers will be things like microscopes and perhaps more advanced kind of aids to help us perform the highest quality endodontic treatment*.” (p16f4)<br>"*It's very difficult to be like troughing if you can't see what you're doing so you know*." (p3f1)

Supplementary File 4 Coding from adapted PAcE template: environmental factors

Subcategories	Description	Illustration
**Social and organisational**	Dentists are not adequately paid under the NHS. There has been no change in remuneration system from past.	"*I qualified in 2001 and what we get paid since then hasn't really increased in line with the increasing cost of everything else or equipment, rates, electricity, wages etc.*" (p4f2)<br>"*The financial reward for doing is in my considered opinion appalling for what they're expecting us to or what we are taught as undergraduates*."(p15f4)
	Complexity such as the discovery of additional canal is not recognised in the current fee structure.	*"[…] That there is no system in place for depending how many canals the tooth has as what you get paid […] sometimes in the [NHS] if you find an extra canal or extra 2 canals that makes your job more difficult […] you have to give more time to it but you're still getting remunerated in the same way"* (p1f1)
	The practitioners who work on salaries in hospital are not driven by how much they get for treatments.	"*And obviously within academic practice or clinical practice within hospital where I'm salaried and therefore you know it's driven by patient need and I know there's no questions in your head about how much you're taking home for the treatment*" (p3f1)
	Supportive environment encourages use of rubber dam in general dental practice.	"*It's definitely environment, If it is supportive and it encourages its [rubber dam] use*" (p3f1)<br>"*the first practice I worked in as a VT practice with a rubber dam kit It wasn't really…, It was actively discouraged and believe it or not when I moved practice four years later? I knew that this was something I should be doing*" (p6f2)

	The practices might be very reluctant in buying instruments and equipment's due to expenditures.	"*I think is it sometimes practice owners could be little bit short sighted in investing because they don't think about the long-term effects of their investment*." (p3 f1)<br>"*In practice you're very much constrained by what the principal buys. Unless you go in and self-invest which I find very upsetting*." (p15f4)
	The referral system in NHS is very difficult and time taking and if the cases are rejected by the system, it is hard for dentists to achieve adequate results because of the complexity of the treatment.	"*[…] the process of trying to get that patient referred was very long and painstaking. To be honest it was a continual back and forth and with the end gateway*" (p16f4)<br>"*[available resources or support on specialist level in NHS] I think that's part of problem because you end up taking cases that you may think that slightly more complicated than you would like to be doing, which will probably affect your outcome*" (p1f1)<br>"*[..]And obviously the referral process is from my own experience as part, I haven't found it was easiest way to kind of have somebody referred to the NHS Gateway. That's only kind of past potential barrier via the NHS route*". (p16f4)
**Education, training and supervising**	Undergraduate training is good as it relates to theory and core knowledge	"*All that can be done in undergraduate education is to deliver the core knowledge*." (p3f1)<br>"*I think the endodontic training that our undergraduate colleagues get now is infinitely better than what I received when I was undergraduate*" (p15f4)
	There is lack of volume in undergraduate training.	"*I think the barrier we have for undergraduate teaching this amount of exposure they get to cases*" (p4f2)

	Training is required by the dentists in order to improve and enhance their skills and it is essential to educate practitioners with advance knowledge.	"*It's [postgraduation] mandatory that we do continually revisit refresh and go back to hearing even going back to hearing the basics again. I think that's just part of being a professional person[..]*" (p15f4)<br>*"Post-graduation you will always need to do some additional learning and update and there's always going to be some form of updates and techniques or methods things. So, there will always be part of personal things[….]"* (p16f4)
	Training dentist to specialists' level might improve the quality of the treatment across the whole system.	"*I think if you really want to improve the quality of endodontics it's gotta be dragged up from the top, you have to train more specialists and have endodontic practices.*" (p11f3)
**Policy level, government, cultural, regulatory influence**	A network of dentists working in different complexity levels of care based on their training will improve patient care.	"*I would like to think of is more of a kind of managed clinical network actually and encourage there to be dentists with special interests within NHS general practice who focus on endodontics so that other, General dental practitioners who are not quite as comfortable in endodontics refer to them*". (p9f3)

Interview questions:

1. How good would you say endodontic treatment in your practice is?
2. Are there any barriers to carrying out optimal Endodontic treatment in your practice?
    - i. Do you think that you are adequately remunerated for carrying out endodontic treatment?
    - ii. Do you think that the time necessary to complete good quality endodontic treatment affects your willingness or ability to provide this treatment?
    - iii. Do you think that you have had the necessary training to carry out multirooted endodontic treatment to a high standard?
    - iv. In your opinion is it feasible for GDPs to have all the necessary equipment to carry out routine good quality endodontic treatment?
    - v. Do you feel that there are sufficient resources available to support you in the management of difficult endodontic anatomy?
3. Do you think that post graduate training is required to enable dentists to carry out good quality root treatment? In your opinion/experience do undergraduate dentists receive sufficient training in endodontics?
4. How does patient attitude and expectation affect your decision making in endodontics treatment?
5. Anecdotally rubber dam use in endodontics does not appear to be universal.
    - i. What do you think the reasons for this are?
    - ii. What might increase uptake?
6. How has the COVID pandemic affected your practice of endodontics?

I. How has the COVID-19 pandemic affected your attitude to tooth retention using root treatment.

II. How has the COVID-19 Pandemic affected your access to support with difficult root treatments?

7. What if anything would make it easier to do good quality endodontic treatment in your practice?

Suggestions to improve the quality of care

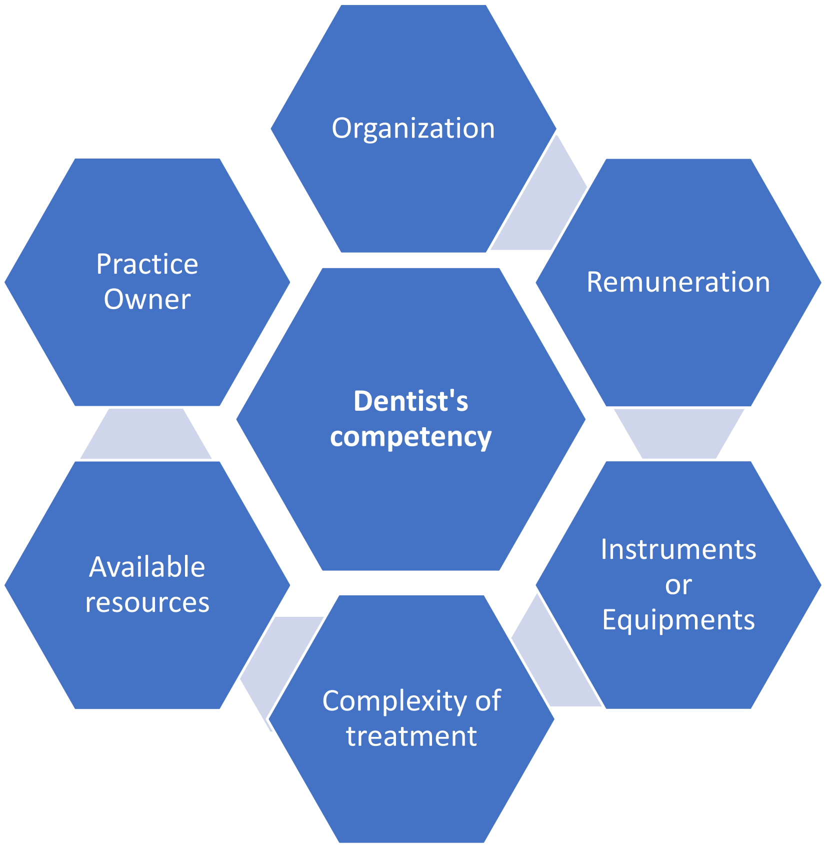

Supplement: Supplementary file 1 — Supplementary Information (PDF 127KB) [file 41415_2022_4475_MOESM1_ESM.pdf]
